# ﻿Redescription of the enigmatic myriapod *Hanseniellachilensis* (Hansen, 1903) (Symphyla, Scutigerellidae) based on scanning electron microscope images of Chilean specimens

**DOI:** 10.3897/zookeys.1198.119723

**Published:** 2024-04-22

**Authors:** Antonio Parra-Gómez, Jorge Pérez-Schultheiss, Leonardo D. Fernández

**Affiliations:** 1 Facultad de Ciencias, Universidad Austral de Chile, Av. Rector Eduardo Morales Miranda 23, Valdivia, Chile; 2 Área Zoología de Invertebrados, Museo Nacional de Historia Natural, Casilla 787, Correo Central, Santiago, Chile; 3 Programa de Pós-Graduação em Biologia Animal, Instituto de Biociências, Universidade Federal do Rio Grande do Sul. Porto Alegre, Rio Grande do Sul, Brazil; 4 Departamento de Zoologia (Laboratório de Carcinologia), Instituto de Biociências, Universidade Federal do Rio Grande do Sul. Porto Alegre, Rio Grande do Sul, Brazil; 5 Facultad de Medicina Veterinaria y Agronomía, Universidad de Las Américas, Manuel Montt Nº 948, Providencia, Santiago, Chile; 6 Centro de Investigación en Recursos Naturales y Sustentabilidad (CIRENYS), Universidad Bernardo O’Higgins, Av. Viel 1497, Santiago, Chile

**Keywords:** Biodiversity hotspot, biogeography, Chile, garden centipede, Myriapoda, pseudocentipede, SEM images, soil-dwelling arthropod, symphylans, taxonomy

## Abstract

*Hanseniellachilensis* is the only myriapod of the class Symphyla known from Chile. This garden centipede, or pseudocentipede, was described more than 120 years ago based on morphologically incomplete specimens collected in central Chile, a well-known biodiversity hotspot. In this study, we redescribe this species based on morphologically complete specimens collected near the type locality using scanning electron microscope images. Our study provides the description of diagnostic characters hitherto unknown in this species such as macrochaetae of the tergites and spinnerets of the cerci. We also include a new record from central Chile and discuss the presumed presence of this species in Argentina and Madagascar.

## ﻿Introduction

Symphylans, also known as garden centipedes or pseudocentipedes, are soil-dwelling arthropods of the class Symphyla in the subphylum Myriapoda. Symphylans are among the least studied soil microarthropods in the world. Often, the identity and geographic distribution of these myriapods are uncertain because they are relatively small and so difficult to study that many researchers simply overlook them (Minelli and Golovatch 2001). Globally, these invertebrates are represented by around 250 species and two families, namely Scutigerellidae and Scolopendrellidae (Minelli 2011; Salazar-Moncada et al. 2015; Jin and Bu 2020; Jin et al. 2023). Symphyla inhabit moist soils on all continents except Antarctica (Minelli 2011). They are whitish and fragile-looking, typically measuring between 5 and 8 mm in length (Minelli and Golovatch 2001).

In the Neotropical region, symphylans are represented by 21 species distributed across six genera and two families (Scheller and Adis 1996). Of these 21 species, only 14 are endemic to this biogeographic region. The known number of Neotropical species is remarkably low considering the area and habitat heterogeneity of the region (Scheller and Adis 1996). These 14 species likely represent a tiny fraction of the true and still unknown diversity of Neotropical symphylans.

Chilean symphylans are among the least known in the Neotropical region (Vega-Román et al. 2012). In 1897, Attems reported the presence of *Scutigerellaimmaculata* (Newport, 1845) on Navarino Island, a remote island on the southern tip of South America, Chile, between the Beagle Channel and the Drake Passage (Attems 1897). However, *S.immaculata* is endemic to the Holarctic region (northern hemisphere) and, therefore, Scheller (1992) suspects that Attems may have observed a similar, yet unidentified species on Navarino Island. Two years later, Silvestri (1899) also reported *S.immaculata* in central Chile, a well-known biodiversity hotspot. However, shortly afterwards, Hansen (1903) concluded that the specimens observed by Silvestri belonged to a new species he called *Scutigerellachilensis* Hansen, 1903. Finally, Bagnall (1913) transferred *S.chilensis* to the genus *Hanseniella*. Currently, *Hanseniellachilensis* (Hansen, 1903) is the only known symphylan species for Chile.

Later, Aubry and Masson (1953) allegedly recorded *H.chilensis* in Madagascar, which is more than 11,000 km from the type locality (central Chile) of this species. *Hanseniellachilensis* has not been observed again on that island, so its occurrence in Madagascar has yet to be confirmed. Subsequently, Juberthie-Jupeau (1962) documented the presence of *H.chilensis* in Argentina. This researcher noted that the specimens observed in Argentina were about twice as long as the specimens described in Chile.

To date, it is unknown whether the symphylans recorded in Madagascar and Argentina belonged to *H.chilensis* or to other similar yet undescribed species. Unfortunately, Hansen’s original description of *H.chilensis* was based on individuals that lacked key morphological traits for identification and diagnosis of symphylans, such as most macrochaetae of the tergites and spinnerets of the cerci (Hansen 1903). Therefore, it is necessary to redescribe *H.chilensis* based on complete specimens and modern methods to improve the identification of this species and our knowledge of Neotropical symphylans.

In this study, we redescribe *H.chilensis* based on scanning electron microscope images of morphologically complete specimens collected in central Chile. Additionally, we report a new record in Chile and discuss the presumed presence of this species in Madagascar and Argentina.

## ﻿Material and methods

Specimens of *H.chilensis* included in this study were collected between 2022 and 2023 in the rural locality of Lefuco, near Temuco city, La Araucanía region, central Chile. This reference is important because some of the specimens used by Hansen to describe *H.chilensis* came from the surroundings of this city (Hansen 1903). In the field, individuals were photographed alive with an Olympus Tough TG-6 digital camera, collected by hand and deposited in vials with 95% ethanol. The geographic location of each specimen was recorded using the coordinate format proposed by the world geodetic reference system WGS84. The georeferenced data were plotted on a map constructed with QGIS v. 3.28.1 with ©MapTiler and ©OpenStreetMap data.

Once in the laboratory, specimens were deposited in Petri dishes with 95% ethanol or mounted on concave microscope slides filled with glycerol and examined under dissecting and light microscopes, respectively. Morphological features were measured using IMAGEJ v. 1.53u software (Schneider et al. 2012).

Specimens coded as APG-17-a and PG-71-L-a (field code, see results) were photographed under the scanning electron microscope (SEM). SEM specimens were mounted on stubs and then dehydrated using a Hitachi HCP-2 critical point dryer. Afterwards, specimens were coated with gold and palladium on a Leica EM ACE200 and photographed with a Zeiss EVO M10 SEM operating at 20 kV. The SEM photographs were processed in GIMP v. 2.10.32 software, and FIGUREJ plugin for IMAGEJ (Mutterer and Zinck 2013).

The investigated specimens were deposited at the Museo Nacional de Historia Natural de Chile (National Museum of Natural History of Chile, MNHN), Santiago, Chile.

## ﻿Results

### ﻿Taxonomy

**Subphylum Myriapoda Latreille, 1797**

**Class Symphyla Ryder, 1880**

**Family Scutigerellidae Bagnall, 1913**

**Genus *Hanseniella* Bagnall, 1913**

#### ﻿ Hanseniella chilensis

Taxon classificationAnimaliaScutigeromorpha Scutigerellidae

(Hansen, 1903)

E25FA3B0-2427-5377-BCA7-68925E962337

**Figure 1.**
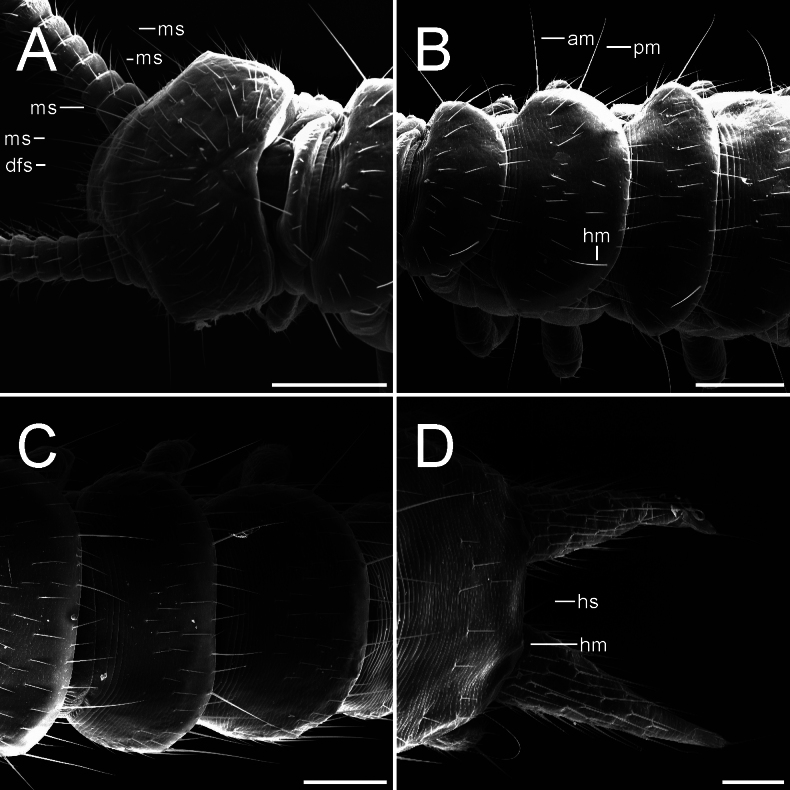
APG-17-a Male, dorsal view **A** head, first tergite and part of the second tergite (dfs-distinct frontal seta, ms-macroseta) **B** tergites 2–5 (am-anterolateral macrochaeta, hm-hind macrochaeta, pm-posterolateral macrochaeta) **C** tergites 11–13 **D** last tergite and cerci (hm-hind macrochaeta (abraded), hs-hind seta). Scale bars: 200.0 µm (**A–C**); 100.0 µm (**D**).

**Figure 2.**
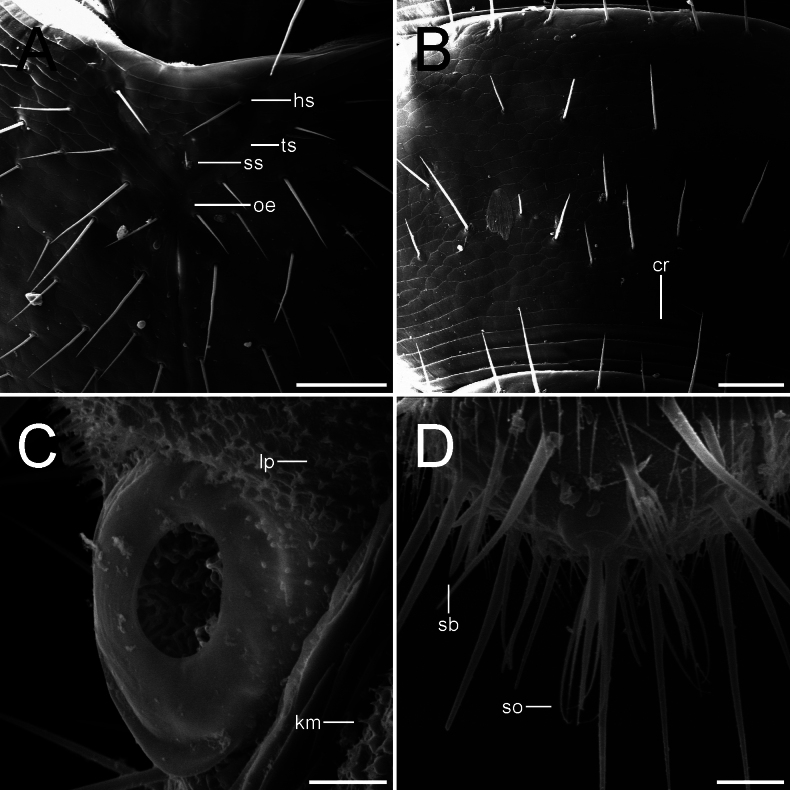
Details of some relevant structures of APG-17-a Male (**A, B**) and PG-71-L-a Female (**C, D**) **A** central rod and triangular sulcus from the top of the head (hs-hind seta, oe-ovoid end, ss-short seta, ts-triangular sulcus) **B** tergal surface of tergite 3 (cr-cuticular rim) **C** Tömösváry organ and proximal surface (km-knob with microseta, lp-linguiform protuberance) **D** apical zone of the last antennomere (sb-sensilla basiconica, so-sensory organ). Scale bars: 50.0 µm (**A, B**); 10.0 µm (**C, D**).

**Figure 3.**
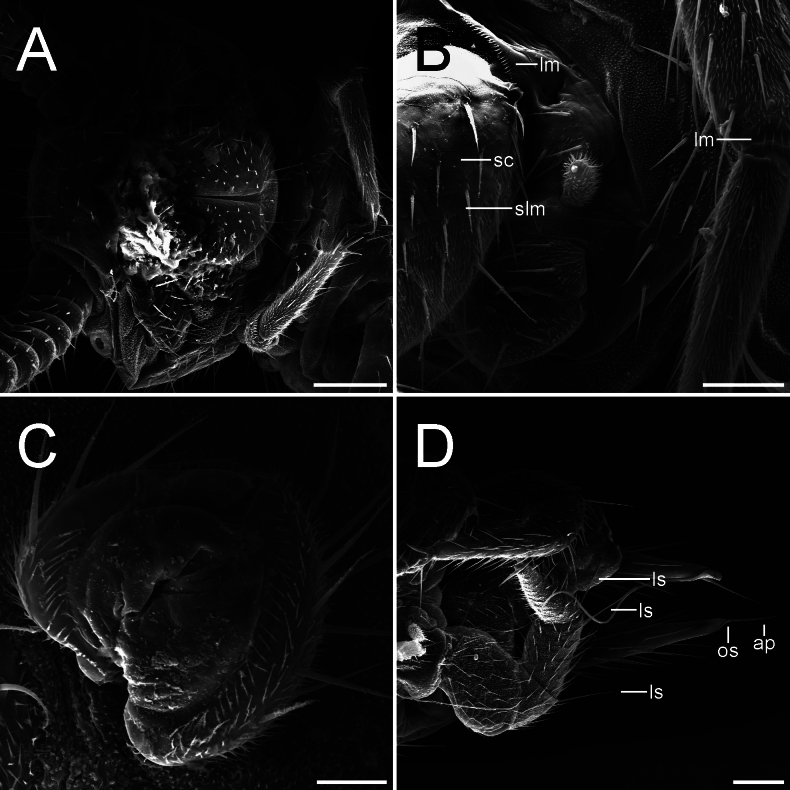
PG-71-L-a Female, ventral view **A** head and first pair of legs **B** ventral surface details proximal to left leg of pair 8 and its 2 first podomeres, podomere 4 and 5 of pair 7 partially show (lm-laminar needles, sc-scale-like cuticle, slm-“scales” with laminar needles) **C** coxal sac near leg pair 4 **D** last pair of legs and cerci (ap-apical seta, ls-large seta, os-outer seta). Scale bars: 100.0 µm (**A, D**); 50.0 µm (**B**); 20.0 µm (**C**).

**Figure 4.**
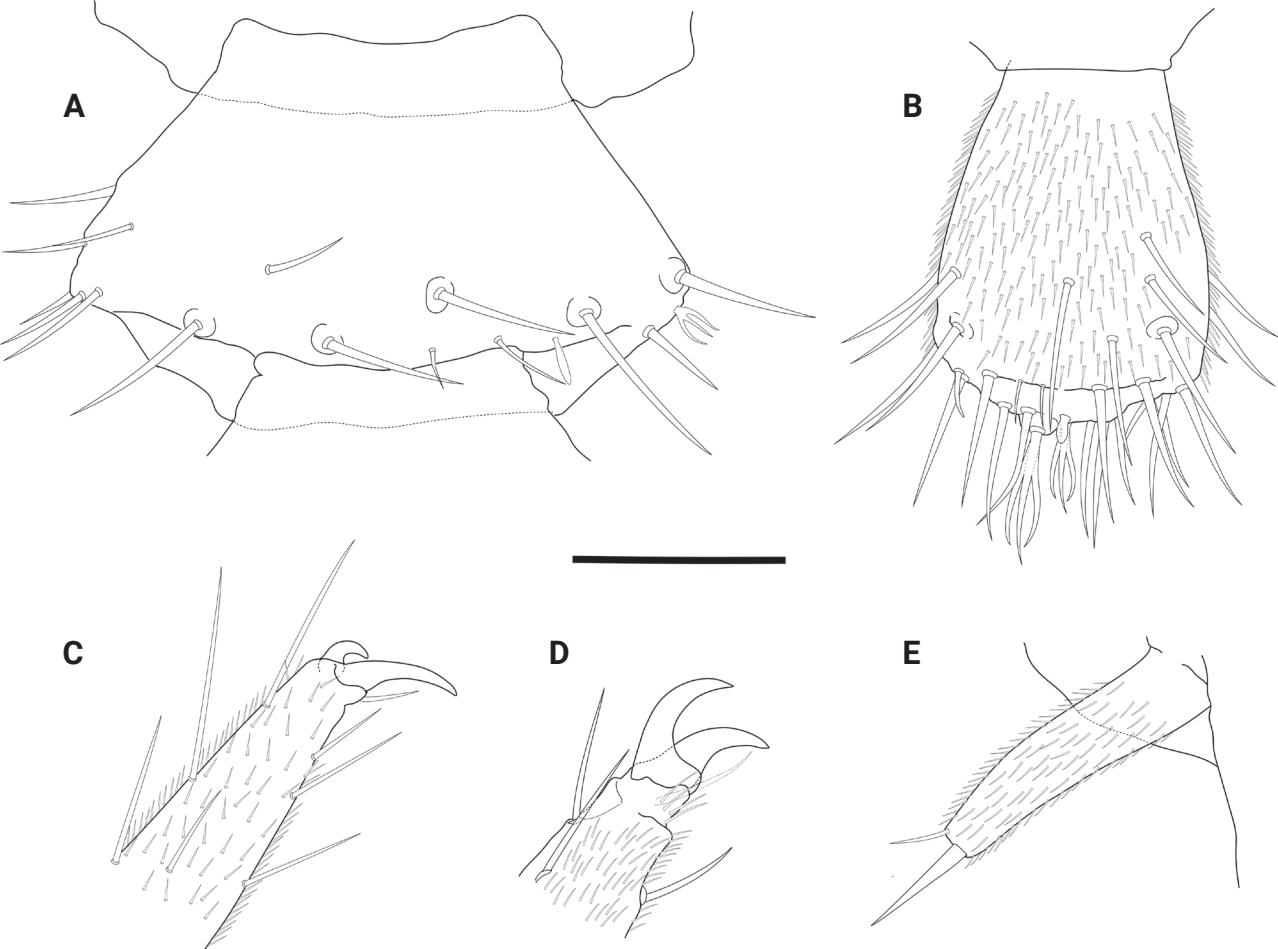
Illustrations showing some relevant structures of the species **A** antennomere 20 of a male **B** apical antennomere (sensory organs not as detailed as described due to their small size, smallest one not visible on this angle) **C** distal end of the first pair of legs **D** distal end of the last pair of legs (frontal seta abraded) **E** styli. Scale bar: 50 µm.

**Figure 5.**
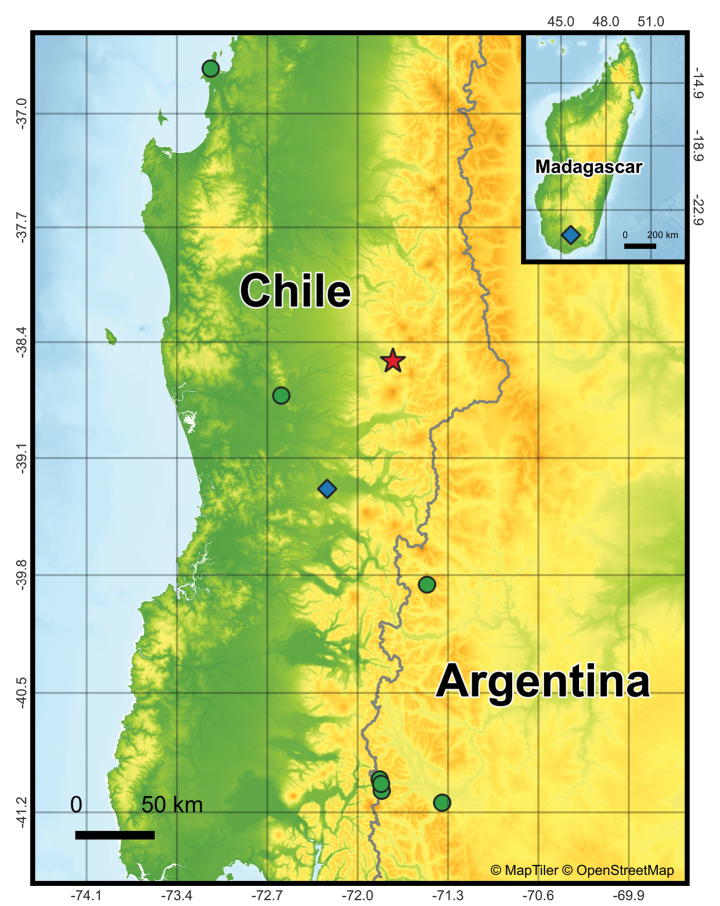
All known records of *Hanseniellachilensis* (Hansen, 1903). Diamond = uncertain records from the literature, Star = new record herein, Circle = literature records.

**Figure 6.**
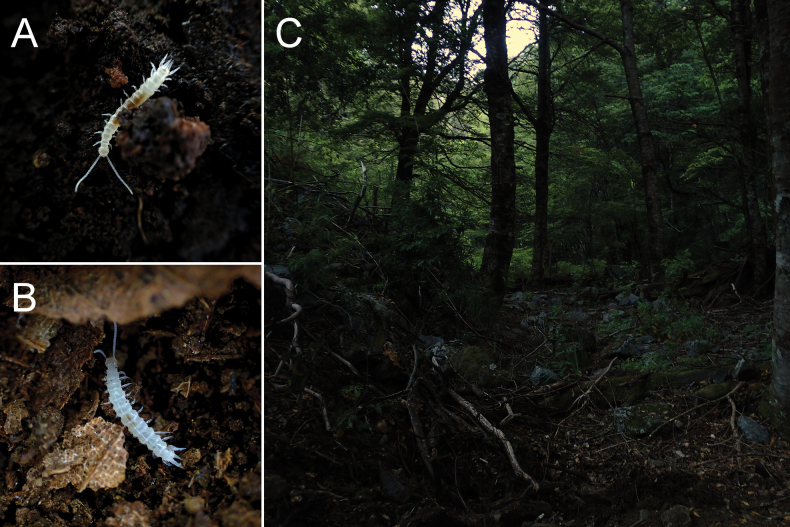
**A**, **B** habitus of the species **C** general habitat where the species can be found.

Figs 1 , 2 , 3 , 4 , 5 , 6

Scolopendrella immaculata Silvestri 1899: 370 (Not Newport, 1845).
Scutigerella chilensis Hansen, 1903: 27, 32, 46, 48, 51, plate 4, figs 4a–g; Silvestri 1905: 746; Porter 1912: 52; Domínguez 1992: 40; Vega-Román et al. 2012: 21, fig. 1.
Hanseniella (Hanseniella) chilensis Bagnall 1913: 198.
Hanseniella chilensis Ringuelet 1955: 110; ?Aubry and Masson 1953: 63; ?Juberthie-Jupeau 1962: 63, 75, fig. 5b; Scheller 1979: 607; 1992: 171; Soesbergen 2019: 34.

##### Type locality.

Types not designated by Hansen (1903), but the author described species based on specimens collected from San Vicente, Biobío region and Temuco, La Araucanía region, central Chile.

##### Material studied.

2 males, **Chile**: La Araucanía region, Malleco province, Estero Lefuco, under rotting wood chips, -38.5153, -71.7273, 15-I-2022, APG14 (field code); 1 male, same locality, leaf litter, -38.5135, -71.7263, 31-I-2022, APG17-a (field code); 1 male, 2 female, same locality, under a rotting log ca. 944 m a.s.l., -38.5132, -71.7275, 19-IX-2023, PG-71-L/PG-71-L-a (field code).

##### Diagnosis.

Adults specimens of *Hanseniellachilensis* can be separated from related species by the following combination of characters: Central rod follow by a triangular sulcus with a distinct small anterior seta, and two posterior setae (Figs 1A, 2A); antennae usually with 29–37 (30–40 in Hansen (1903) specimens) antennomeres; first tergite rudimentary with one row of 9–11 setae (Fig. 1A); dorsal cuticle scale-like and no pubescence or microsetae present (Fig. 2B), macrochaetae present on most tergites as in Table 1 (Fig. 1B–D); first podomere of first pair of legs bearing a posterolateral line of ca. 10 laminar needles; third podomere of 12^th^ pair with one large dorsoposterior seta, 0.8 times the breadth of the podomere and fourth podomere bearing two distinct dorsoposterior larger setae, the largest being 1.2 times the breadth of the podomere [larger than in *Hanseniellacapensis* (Hansen, 1903)].

**Table 1.** Chaetotaxy and posterior margin shape of the tergites, except the rudimentary first tergite.

Tergite number	Number of setae on tergal surface and margins (ca.)	Number of rows (counting hind setae as so)	Anterolateral macrochaeta (per side)	Posterolateral macrochaeta (per side)	Hind macrochaeta pointing outwards and/or forwards (per side)	Posterior margin
2	33–38	3	1	1	1	Almost straight
3	41–55	3	1	1	1	Almost straight
4	42–52	3	0	1	1	Almost straight
5	37–47	3	0	1	1	Almost straight
6	61–82	5	1	1	1	Almost straight
7	56–65	3	0	1	1	Slightly concave
8	38–55	3	0	1	1	Slightly concave
9	67–82	5	1	1	1	Almost straight
10	57–65	3	0	1	1	Slightly concave
11	44–59	3	0	1	1	Slightly concave
12	66–84	5	1	1	1	Almost straight
13	54–65	3	0	1	1	Slightly concave
14	61–71	5	0	1	1	Slightly concave
15	33–46	2–3	0	0	1	Convex between cerci

##### Description.

Length of body (measured dorsally) without cerci and antennae: female ca. 3.5–4.3 mm, males ca. 4.7–5.2 mm. ***Head*.** 1.4 times broader than long, frontal margin slightly convex with 1+1 distinct setae, lateral margins convex with a sharp anterolateral angle, posterior margin concave with rounded posterolateral angles (Fig. 1A). Central rod well defined, posterior end slightly ovoid, followed by a triangular sulcus with a short seta near the anterior end, and 1+1 hind setae, both directed inwards and forwards (Figs 1A, 2A). Frontal and top of head sparsely covered with setae, 3+3 macrosetae near the antennal base and 1+1 anterolateral macrosetae, ca. 2.1 as long as common head setae (Fig. 1A). Tömösváry organ circular, proximal surface covered by linguiform protuberances and small knobs bearing microsetae (Fig. 2C). First maxillae simple, with a single subtriangular palp, ca. 1.6–2.0 longer than wider, around the same length of the most proximal setae. Second maxillae distal margin with 3+3 papilla-bearing lobes, anterior part with several protuberances, each one supporting a single seta, anterolateral margin with 3+3 (some males with 4+4) two-forked organs with a small medial process subtruncated at the tip. Both maxillae bearing setae across the surface. ***Antennae*.** Long, ca. 0.4–0.5 times the body length, with 29–37 antennomeres. Surface covered by pubescence, 1^st^ antennomere with only one distal whorl of setae, 2^nd^–10^th^ antennomeres with two poorly defined whorls very close to each other, middle whorl composed by larger setae and the distal by shorter setae, a 3^rd^ whorl begins around 8^th^–14^th^ antennomere below middle whorl. Small tri-forked organ on the distal margin starting from 4–9^th^ antennomere and on, appears to be four-forked on later antennomeres. Sensilla basiconica present on distal margin from 8–10^th^ antennomere and on, increasing in number and more acuminated in shape towards distal antennomeres (Fig. 4A). Distal margin of antennomeres with granular surface. First antennomeres ca. 1.7–1.8 times broader than long (Figs 1A, 3A), 2–4 antennomeres ca. 2.7–2.8 times broader than long, middle antennomeres ca. 1.1–1.5 times broader than long, distal antennomeres elongated and ca. 0.8 times broader than long. Apical antennomere spherical, sensilla basiconica present, apex of the segment bearing a large sensory organ borne from a small protuberance, composed of a central stalk which yields 5 slightly longer spiniform processes with curved-inwards tips; two additional similar organs, a large and a smaller one, composed of 4 spiniform processes in total besides the central stalk, basal protuberance absent or remarkably reduced (Figs 2D, 4B). ***Tergites*.** Cuticle scale-like, surface smooth except for seta and macrochaeta (Figs 1B–D, 2B). Rows and number of setae as in Table 1. First tergite rudimentary, with one row of 9–11 setae, a pair longer than the rest (however it seems to be a variable character) (Fig. 1A). Anterior surface portion from the second tergite and on with a set of circular cuticular rims (Fig. 2B). Hind setae rather large, increasing gradually but considerable in length towards the posterior body portion, on last segments almost equal in length to macrochaetae (Fig. 1C). First, third, sixth, tenth, and fourteenth tergite semicircular, fourth, fifth, seventh, eleventh, and twelfth subtrapeziform, eighth and thirteen subrectangular, fifteenth subquadrate. Third, sixth, ninth, twelfth and fourteenth tergites longitudinally broader than preceding ones. Posterior margin as in Table 1. Tergal surface with poorly defined rows of setae as in Table 1 (Figs 1B, C, 2B). Second and third tergite bearing on each side one anterolateral macrochaeta directed slightly forwards, one posterolateral macrochaeta directed outwards and forwards, and a posterior macrochaeta borne from the hind margin also directed outwards and forwards (Fig. 1B). Fourth and fifth tergite bearing only one posterolateral and hind-borne macrochaetae on each side (Fig. 1B). Sixth with macrochaetae same as second and third tergite. Seventh and eighth with macrochaetae same as fourth and fifth tergite. Ninth macrochaetae same as sixth tergite. Tenth and eleventh macrochaetae same as seventh and eighth. Twelfth macrochaetae same as ninth. Thirteenth and fourteenth macrochaetae same as tenth and eleventh. Posterior margin of last tergite with two short hind setae between cerci and two macrochaetae pointed outwards near the cerci base, U-shaped incision absent (Fig. 1D). ***Ventral surface*.** Covered by microsetae (Fig. 3B), last segment surface with laminar needles borne at the posterior end of scale-like layers. ***Coxal sacs*.** Mostly heart-shaped, fully developed at the bases on legs 3–9, margins with short setae and around 8–13 larger setae (Fig. 3C). ***Male organs*.** Two very simple ventral contiguous semicircular plates held closely together and covered by pubescence. ***Legs*.** First pair of legs composed of 4 segments, from proximal to distal: First podomere short, bearing 3–4 setae, lateral cuticle scale-like, bearing a posterolateral line of ca. 10 laminar needles (similar to Fig. 3B). Second podomere 2.0 times longer than wider, dorsal and lateral cuticle scale-like (similar to Fig. 3B), one large distinct seta held proximolaterally and ca. 12 lateral setae, anteroventrally with 2 setae, largest seta held medioventrally and 0.8 times the breadth of the podomere, followed by a short spine held on a small bump, and two ventrodistal large setae, a line of several laminar needles near the laterodistal margin and most distal “scales” also bearing laminar needles (similar to Fig. 3B). Third podomere subequal in length and width, surface pubescent, bearing 3 dorsal setae and 4 lateral setae. First to third podomere distal margins glabrous and microgranulated, scale-like surface also glabrous. Fourth podomere 3.9 times longer than wider, surface pubescent, with ca. 11 dorsal, 6 lateral and ca. 9 ventral setae; dorsal and ventral setae increasing gradually in length towards the apex. Two claws, posterior more curve and around 3/5 the length of the anterior claw, frontal seta around 1/2 the length of the anterior claw (Figs 3A, 4C). 12^th^ pair of legs composed of 5 segments: First podomere short, with ca. 12–13 ventral setae, surface scale-like, distal “scales” bearing laminar needles. Second podomere 1.7 times longer than wider, dorsal surface scale-like, 1–3 distodorsal setae, ca. 31 lateral setae, dorsolateral surface also scale-like, lateroventrally with “scales” bearing laminar needles (similar to Fig. 3B), and a short spine near the margin held on a small bump, ventral surface pubescent and bearing ca. 14 setae, 2 large distinct setae and one medial short spine. Third podomere subequal in length and width, bearing ca. 13 dorsal setae and one large dorsoposterior seta, 0.8 times the breadth of the podomere (Fig. 3D), laterally with ca. 9 setae, posterior margin with a line of several needles and “scales” also with laminar needles. Fourth podomere 1.6 times longer than wider, surface pubescent, bearing several setae and two distinct dorsoposterior larger setae, the largest being 1.2 times the breadth of the podomere (Fig. 3D). First to fourth podomere distal margins glabrous and microgranulated, scale-like surface also glabrous. Fifth podomere 3.1 times longer than wider, surface pubescent with several setae. Dorsal setae of podomeres generally longer than the ventral ones (Fig. 3D). Two claws, both curve, anterior claw basally thicker and slightly longer, frontal setae around 7/10 the length of anterior claw (Fig. 4D). ***Styli*.** Short and straight, densely covered by pubescence, bearing two distinct large setae on distal end, largest one around 1.8–2.2 times the length of the shorter one, last one difficult to see sometimes, held posterolaterally (Fig. 4E). ***Sense calicles*.** Pit margin covered by simple short setae and what appears to be bi- and tri-branched setae. Two distinct larger setae posteromedial to pit, 5.3 times the length of the short pit margin setae. Sensory seta inserted in the middle of the cavity, very long. ***Cerci*.** Surface covered by medium-size setae, which increase in length towards the apex, distal end without setae, apical seta 0.9 times the wider part of cerci, accompanied by a smaller outer seta around 0.25 times the length of the apical seta (Figs 1D, 3D).

##### Distribution.

Africa: ?Madagascar: Banks of Betaly River, near Bezavona (see Remarks); South America: Argentina: Neuquén Province: Lago Curruhué (Currhue mendum Juberthie-Jupeau 1962); Lago Los Cántaros. Neuquén-Río Negro Provinces: Nahuel Huapi Reserve. Río Negro Province: Lago Frías; Puerto Blest. Chile: Biobío Region: San Vicente. Araucanía Region: Lefuco; Temuco; ?Villarrica (see Remarks). (Silvestri 1899; Hansen 1903; Aubry and Masson 1953; Juberthie-Jupeau 1962) (Fig. 5).

##### Remarks.

Direction of macrochaetae can be variable due to specimen conservation and preparation on slides, but on live specimens in the field, the macrochaetae seem to point forwards in most, if not all, tergites (Fig. 6A–C); it is important to note that some setae and macrochaetae were abraded on the SEM specimens. An additional record of *H.chilensis* is reported in Madagascar by Aubry and Masson (1953), however, it is very likely that it is a morphologically similar species or accidentally introduced on the island; the authors likewise point out that the presence of this species on the island is strange (Fig. 5). Silvestri (1899) also mentions Villarrica as an additional record where he observed *H.chilensis* (at the time misidentified as *S.immaculata*), nevertheless, this locality is never mentioned again by Hansen (1903) nor Bagnall (1913) (Fig. 5).

##### Affinities.

Investigation of complete specimens of *H.chilensis* collected in central Chile allowed us to conclude that this species shares important morphological similarities with only one other congeneric species. Particularly, we noted that *H.chilensis* exhibits macrochaetae on all tergites (excluding the rudimentary first), a trait shared only with *H.capensis* (Hansen 1903; Soesbergen 2019). Although it can be easily distinguished from *H.capensis* by the shape of the claws of the last pair of legs, as in this last species, the claws are distinctively thicker basally (Hansen 1903). Also, the overall number of macrochaeta per tergite is higher in *H.chilensis* than in *H.capensis*.

Additionally, Soesbergen (2019) described in his appendix that *H.arborea* Scheller, 1979 possesses macrochaeta on all tergites; however, in the original description, Scheller (1979) explicitly describes that the species exhibit macrochaeta on tergites 2–4, 6, 7, 9, 10, 12, 13 and 14. This last author also mentions that the distribution of macroseta or “large setae” on the fourth and fifth podomeres of the last pair of legs is closely similar to *H.chilensis*, but we noted that they are remarkedly shorter in length.

Unfortunately, we were unable to compare *H.chilensis* with *H.hardyi* (Chamberlin, 1920), *H.neozelandica* (Chamberlin, 1920) and *H.paolettii* Scheller, 1993 because the number of macrochaetae present in their tergites remains unknown (Soesbergen 2019). Therefore, the morphological affinity between *H.chilensis* and these last three species remains mostly unresolved.

## ﻿Discussion

*Hanseniellachilensis* is a symphylan species described by Hansen (1903) using damaged specimens collected in central Chile. Our study provides a detailed redescription of this species based on morphological analysis via SEM images and microscopy observation of morphologically complete specimens. We collected our specimens in Lefuco, a rural locality near Temuco, the place of origin of some of the specimens used by Hansen to describe *H.chilensis* in 1903. One of the most important contributions of our study was to describe, for the first time, morphological traits that were previously unknown in this species, such as the macrochaetae of all tergites. These traits were not included in the original description of *H.chilensis* and remained unknown for more than 120 years. The lack of knowledge of these and other morphological traits has historically hindered the identification of this species.

After being described in Chile, *H.chilensis* was recorded in Madagascar (Aubry and Masson 1953) and in Argentina (Juberthie-Jupeau 1962). At present, it is not clear whether the symphylans recorded in Madagascar and Argentina really belong to *H.chilensis*. Prior to our study, some important diagnostic characters of this species were unknown, and therefore, there is a high chance that specimens identified outside Chile may have belonged to another species.

The latter scenario is plausible because Chile is surrounded by biogeographic barriers that limit species dispersal, including the Atacama Desert in the north, the Andes Mountains in the east, the Pacific Ocean in the west, and the Drake Passage to the south (Fernández et al. 2016). These barriers have kept Chilean biota isolated from the rest of the world for thousands of years and have favored the radiation of endemic animals, plants and microorganisms (e.g., Eisenberg and Redford Kent 1992; Lazo Araya 2015; Fernández et al. 2015, Rodriguez et al. 2018; Campello-Nunes et al. 2022; Parra-Gómez and Fernández 2022). These barriers surely represent insurmountable hurdles for animals such as symphylans. It is difficult to imagine them climbing mountains of more than 6,000 m altitude to cross from Chile to Argentina or swimming more than 11,000 km to reach Madagascar. At least not by their own means. Therefore, *H.chilensis* could be a species endemic to central Chile, a region recognized as a biodiversity hotspot.

Alternatively, *H.chilensis* could be a truly ubiquitous species, which has managed to establish viable populations in different countries. The means of dispersal of symphylans remains unknown, but we can assume that they could overcome biogeographic barriers by passive dispersal. Airborne dispersal is used by some arthropods, but symphylans are unlikely to use this method. They are soil-dwelling and apparently do not have morpho-physiological adaptations to balloon-like spiders or to resist desiccation and UV radiation for long periods of time. Phoretic dispersal seems more plausible since symphylans could overcome biogeographic barriers by being transported on the fur of mammals or feathers of migratory birds. They could also colonize islands by floating on objects such as driftwood. Accidental introduction might be another plausible means of passive dispersal. There is currently an active trade in raw materials and products of plant origin among Chile, Madagascar and Argentina (World Trade Organization 2024). Repeated introductions of *H.chilensis* could eventually favor the establishment of viable populations in exotic sites.

The ecology of *H.chilensis* is unknown, so we are unaware of the consequences that the introduction of this species into exotic ecosystems could have. For example, the symphylan *Scutigerellaimmaculata* has been accidentally introduced in several countries and now is considered an agricultural pest (Waterhouse 1968). Moreover, we ignore the geographic origin of this species. If we assume that *H.chilensis* is a ubiquitous species it could have radiated and been introduced in any of the countries where it has been reported. Perhaps the symphylans identified as *H.chilensis* in Chile (Hansen 1903), Madagascar (Aubry and Masson 1953) and Argentina (Juberthie-Jupeau 1962) are just ecophenotypes of the same species, which could explain why Argentine specimens are longer than those from Chile.

The information provided in our study could contribute to resolving the geographic range of *H.chilensis*, including its status as an endemic or ubiquitous species. We have redescribed *H.chilensis* in detail: we provided SEM images as well as drawings and descriptions of diagnostic characters that were previously unknown in this species. Therefore, it is now possible to investigate whether the putative specimens of *H.chilensis* from Madagascar and Argentina belong to the same species. The resolution of this long-standing question would not only improve our understanding of Symphyla diversity, but also provide indirect information on the dispersal strategies and dispersal ability of these arthropods.

## Supplementary Material

XML Treatment for Hanseniella chilensis

## ﻿Additional information

### Author ORCIDs

Antonio Parra-Gómez https://orcid.org/0000-0002-4144-6610

Jorge Pérez-Schultheiss https://orcid.org/0000-0003-4537-5677

Leonardo D. Fernández https://orcid.org/0000-0001-9550-1921

### Data availability

All of the data that support the findings of this study are available in the main text.
